# Developing and Pretesting a Text Messaging Program for Health Behavior Change: Recommended Steps

**DOI:** 10.2196/mhealth.4917

**Published:** 2015-12-21

**Authors:** Lorien C Abroms, Robyn Whittaker, Caroline Free, Judith Mendel Van Alstyne, Jennifer M Schindler-Ruwisch

**Affiliations:** ^1^ The Milken Institute School of Public Health The George Washington University Washington, DC United States; ^2^ National Institute for Health Innovation The University of Auckland Auckland New Zealand; ^3^ Clinical Trials Research Unit London School of Hygiene and Tropical Medicine London United Kingdom

**Keywords:** mHealth, telemedicine, SMS, text messaging, behavior change, behavior modification

## Abstract

**Background:**

A growing body of evidence demonstrates that text messaging-based programs (short message service [SMS]) on mobile phones can help people modify health behaviors. Most of these programs have consisted of automated and sometimes interactive text messages that guide a person through the process of behavior change.

**Objective:**

This paper provides guidance on how to develop text messaging programs aimed at changing health behaviors.

**Methods:**

Based on their collective experience in designing, developing, and evaluating text messaging programs and a review of the literature, the authors drafted the guide. One author initially drafted the guide and the others provided input and review.

**Results:**

Steps for developing a text messaging program include conducting formative research for insights into the target audience and health behavior, designing the text messaging program, pretesting the text messaging program concept and messages, and revising the text messaging program.

**Conclusions:**

The steps outlined in this guide may help in the development of SMS-based behavior change programs.

## Introduction

A growing body of evidence indicates that text messaging-based programs (short message service [SMS]) on mobile phones can help people modify health behaviors [1-3]. Text messaging programs related to health behaviors have been used in a variety of contexts including HIV prevention, medication adherence, pregnancy education, substance use/smoking cessation, weight loss, diabetes management, and depression [2,3]. Many of these programs have consisted of a series of automated and interactive text messages that guide a person through the process of behavior change.

Text messaging-based programs may be effective for several reasons [1]. Text messages are generally sent by an automated system to the user according to a preset schedule. Studies indicate that text messages are very likely to be read within minutes of being received [4,5]. In contrast to most behavior change programs, they do not require the user to seek out information and support to maintain engagement (eg, by going to a website) [5]. Thus, despite their brevity and text-based limitations, they may have a readership and engagement advantage over other communication modalities [2]. Text messages may be helpful because they provide short, timed bursts of information throughout the day, which are constant reminders of a behavior change goal [5]. This aspect of text messages may maintain goal saliency and confer value beyond the specific content of the behavior change information they contain [3]. Also distinctive of text messaging programs is their ability to provide support and advice in-the-moment or near-the-moment of decision making. This element may be especially important for individuals who are facing strong cravings or recovering from addictions, for which real-time support may make a difference [4]. Finally, text messaging programs that are automated can be designed to mirror elements of in-person counseling, such as by offering tailored advice, behavioral monitoring, goal setting, feedback, and other important behavior change techniques [3,5].

Given the widespread use of texting and mobile phones and the evidence base to support their use, numerous text messaging programs have been developed globally. Current initiatives by the World Health Organization (WHO) such as “Be He@lthy, Be Mobile” and other governmental initiatives exist to address noncommunicable diseases and other health issues worldwide [6]. While there remain privacy concerns associated with text messaging [7], large public health and medical systems in the United States and the United Kingdom have integrated text messaging into their offerings for the public [8,9]. Given the high mobile penetration in the United Kingdom [10], the National Health Service (NHS) rolled out a text messaging program that was integrated into routine clinical services in 2014. The Veterans Health Administration (VHA), the largest health care system in the United States, is in the process of developing a similar program [9]. In addition, in 2013, almost half of US state quitlines that provide phone counseling offered their callers quit smoking text messaging in addition to regular phone counseling services [11,12]. Thus, text messaging has become an acceptable communication platform for achieving public health goals, both in large governmental health systems (ie, NHS and VHA) and in smaller, independent ones.

This paper supports the development of text messaging programs for behavior change and provides guidance on the steps to develop such a program. To date, few published resources exist that describe the process of developing a text messaging program for health behavior change [13,14], and those that do exist are mainly limited to technical aspects of development.

## Methods

The authors drew from the evidence base in recommending specific steps for designing and developing text messaging programs, though this evidence was limited (exceptions include Head et al [3]). Where evidence was lacking, this guide drew on insights gleaned from the collective experience of the authors in designing, developing, and evaluating programs in the United States, United Kingdom, and New Zealand for health behavior change in the areas of smoking cessation, physical activity, healthy eating, and weight management [1,2,5,15-22]. One author (LCA) drafted the guide and the others provided input and review.

## Results

### Steps for Developing a Text Messaging Program

**Figure 1:**
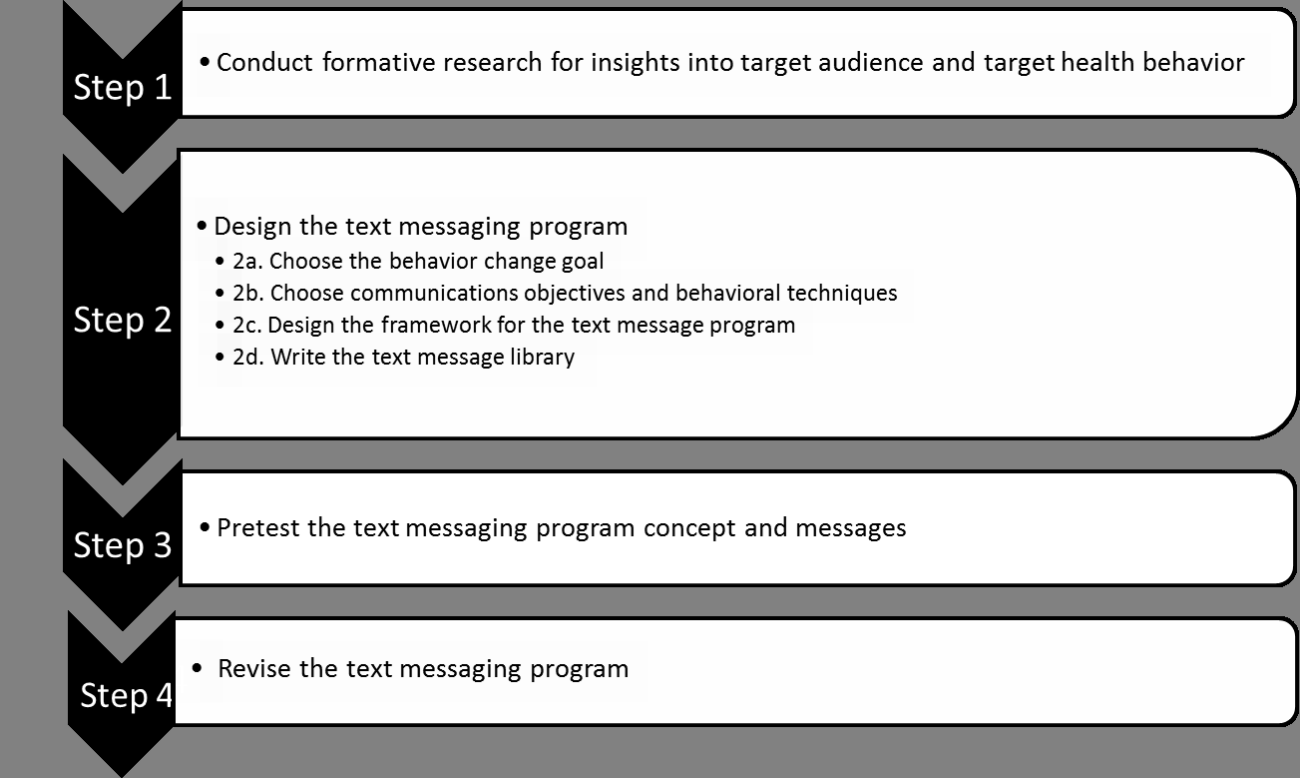
Steps for developing a text messaging program.

Text messaging programs should follow the same phases of development that are typical for any health communication material [23]. As with other programs, once a text messaging program has been developed and pretested, additional evaluation is recommended to determine its efficacy and, after dissemination, its effectiveness. Figure 1 displays the recommended steps for developing and pretesting a text messaging health behavior program [23]. Prior to beginning the design process, formative research should provide the basis for understanding key behavior change mechanisms for the health behavior and population of interest (Step 1). The remainder of this paper will focus on Step 2 (designing the text messaging program) and will also touch on Steps 3 and 4 (pretesting and revising the program).

### Step 2: Designing the Text Messaging Program

#### Step 2a: Choose the Behavior Change Goal and Target Audience

Health behavior change goals should be selected based on a balance of health priorities and characteristics of the target audience, such as readiness to change. For example, the target audience may be pregnant smokers and the behavior change goal may be smoking cessation among those who are interested in quitting. For a more detailed discussion on choosing a behavior change goal and selecting a target audience, see the National Cancer Institute (NCI)’s *Making Health Communication Programs Work* [23]. One consideration that is specific to mobile phones is whether a text messaging program is a good match for the target audience. Factors may include whether the target audience owns a mobile phone, and whether they have a data plan or unlimited text messaging, so that they will not incur charges when receiving messages or responding to the program. For US populations, Pew Research Center’s Internet & American Life Project provides useful information on the digital media habits of segments of the US population, including those segmented by age, gender, ethnicity, and other demographic factors [24]. In the case of pregnant smokers, the ubiquity of mobile phone ownership among women between the ages of 18 and 49 years makes the technology a good fit for the audience [25].

#### Step 2b: Choose the Communication Objectives and Behavioral Techniques

Consideration needs to be given to the communication objectives and behavioral techniques that will be used to promote change in the targeted behavior. Communication objectives generally consist of beliefs, attitudes, and knowledge that people will learn by participating in a given program [23], and behavioral techniques are the actions that people should take to make the targeted behavior change [26]. Communication objectives and behavioral techniques should be based on theory and insights from formative research or other existing research about the factors influencing behavior.

Once communication objectives and behavioral techniques are identified, consideration should be given to how they can be supported by the attributes of text messaging as a modality. Some theoretical constructs may be especially well-suited to mobile communication, such as the Health Belief Model’s construct of cue to action [27] and the use of implementation intentions [28], which prompt the setting of goals around if-then statements (ie, “If I finish work, then I will head to the gym for a workout.”). Likewise, some behavioral techniques, such as tracking of daily goals and receiving feedback on goals, readily lend themselves to mobile programs [26]. For example, in a study about binge drinking behaviors, Suffoletto et al [29] designed their text messages around constructs such as self-monitoring, positive feedback, perceived barriers, and behavioral intentions, drawing from the Health Belief Model, the Information Motivation Behavior Model, and the Theory of Planned Behavior.

**Figure 2:**
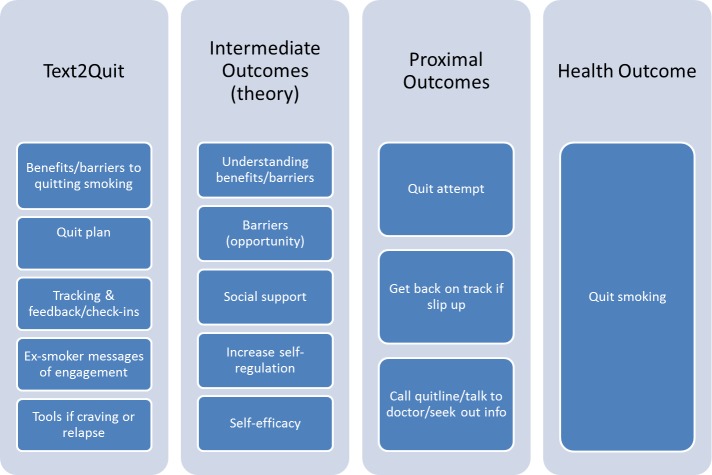
Logic model for Text2Quit, a smoking cessation SMS text messaging program based on social cognitive theory.

We recommend a logic model or behavioral schematic to outline how a particular program’s text message components (inputs) fit with theoretical constructs and proximal and longer-term behavioral and health outcomes. Figure 2 presents a logic model relating the program components of Text2Quit, a smoking cessation text messaging program based on social cognitive theory, to intermediate, proximal, and health outcomes (based on Abroms et al [15]). Text2Quit program components include text messages that contain information on benefits of and barriers to quitting smoking, a quit plan, and an ex-smoker story about quitting from a quitpal, or peer coach. The program also contains text message-based tools for tracking cigarettes smoked and obtaining help if craving cigarettes or smoking. These are hypothesized to affect intermediate constructs, which in turn affect quitting smoking. For example, a person’s understanding of the benefits of and barriers to quitting, sense of social support, self-efficacy, and ability to self-regulate when having a craving are hypothesized to affect proximal outcomes. These may include making quit attempts or calling a quitline, and ultimately impact the person’s likelihood of quitting smoking.

#### Step 2c: Design the Framework for the Program

The framework for the program provides an overarching plan of how messages are sent to users. The framework should include a description of the timing and frequency of messages, as well as indicate the kinds of messages that will “check-in” on users (surveys) and the keywords users will be able to use to ask for additional help in times of need. For an example of a framework and message library, see the QuitNowTxt message library [30]. In designing the framework, decisions may need to be made about the following key issues.

##### Frequency of Messages

The frequency of messages should address the need for the program to communicate key information without overwhelming or burdening the user. Often, text messaging programs send out at least one message/day during key behavior change periods and fewer messages (eg, 3 messages/week) in less acute phases. Depending on the health behavior being targeted, fewer or more text messages per day may be appropriate. For example, Text2Quit sends 5 messages on the quit date, daily messages in the first week after the quit date, and 3 messages per week in the weeks after that [16]. For users who are frequent texters, message frequency may need to be higher so that messages stand out from the many texts they already send and receive daily. It should be noted that some programs only send out texts when a user requests information. For example, SexInfo, a sexual health information service, is a reactive service that replies when the user initiates a question/query to the system [31].

##### Balance of Message Quantity and Importance

The science on message frequency and quantity has not yet identified the ideal “dose.” Poorman et al [32] suggested from a systematic review of the SMS health behavior literature that participants may be better retained if message quantity is varied over time, while Head et al [3] reported that programs with decreasing message frequency were more effective than those with constant message frequencies. A common criticism of these types of programs is that the program sends out “too many messages” [15]. Another related consideration, especially among programs targeting addictive behaviors like eating or smoking, is that the messages may act as a trigger for the behavior that is to be avoided. Abroms et al [15] found that while overall the program was effective, some participants reported that the texts were a trigger for smoking.

##### Timing of Messages

The timing of messages may be related to both the content of the messages (eg, what the messages ask the user to do), the daily routine of the user (eg, when the user is free to consider the text message), and the nature of the behavior change (eg, what time of day is appropriate for targeting that behavior). Consideration also needs to be given to what will trigger the messages (ie, date of enrollment or behavior change, in a weekly cycle). In Papua New Guinea, a text messaging program aimed at providers treating malaria patients was found to be most acceptable in the mornings and during work hours, to help facilitate the usefulness of the reminders [33]. While 2 messages per day were found to be acceptable, providers did not like their repetitiveness over time [33]. A study of binge drinking behaviors concentrated messages in the days leading up to and during the weekends, when binge drinking was more likely to occur [29]. Another study found that adolescent girls preferred the message timing to vary, as it was seen to be less “robotic” if the message timing was unpredictable [34].

##### Nature of Interaction With the Program

Interaction, or bidirectional messaging, is helpful to promote engagement in a program. Shneiderman lists “Eight Golden Rules of Interface design,” of which the following may be especially relevant for SMS programs: provide consistency, offer short-cuts, offer error prevention (ie, “Did you mean this?” or “We didn’t understand you.”) and provide responsive feedback for user-initiated actions [35]. Opportunities for interaction in an SMS program can occur around surveys (eg, “Are you ready to quit? Reply 1 if you are ready or 2 if you are not ready”), with tracking (eg, “How many cigarettes did you smoke yesterday? Reply and see if you met your goal.”) and with keywords. Keywords are words that the user can send into the system at any time for additional help (eg, a user texts the keyword “CRAVE” if having a craving and receives an immediate reply with additional help). Keywords should be limited in number, so that users can easily remember them and use them as needed. By responding to a program survey or using keywords, users can expect to receive positive feedback or an actionable reply from the program. One way to promote interaction is to “gamify” the interaction, providing the user with points that the system can track over time for key types of interaction with the system. Abroms et al used a trivia game as a means of distracting the user during smoking cravings [15]. In Bauer et al’s weight management program [36], responses to check-in questions about eating and physical activity elicited an appropriate automatic response relating to encouragement, motivation, positive reinforcement, or reminders. The algorithms for an automatic text messaging program need to consider human error and include a variety of responses. For example, it might be helpful for the program to recognize common misspellings or typos, so that it will still respond appropriately rather than ignore messages. Regardless of program type, it is important to have an automated message for potential emergency texts and to have protocols in place to manage anticipated or even unanticipated events that may need to be handled by a live person. One solution is to have a real person/provider monitor messages for responses such as, “I have a headache, please help” that the program might not otherwise recognize. Another solution would be to have an automated response that says that the program does not recognize the response and to contact emergency services in the case of a medical emergency. For example, if a person replies to a computer-generated survey with a high-risk response (eg, with a high blood sugar reading or severe weight loss), the response triggers an alert to a clinician who then contacts the patient. In another example, following the release of inpatients from an eating disorders program, one study [37] had staff monitor incoming communication so that if patient responded that they were bingeing, a provider would follow-up with a personalized response rather than allow an automated reply to go through.

##### Source of Messages

The source of text messages is generally the program name (eg, SmokefreeTXT or CDC), so it is easily recognized by the participant as coming from the program. However, automated messages may be supplemented with messages from a real person/counselor/clinician. Even within automated program messages, the message source can vary with some messages coming from the program and others from a specific “person” who is part of the program**.** For example, in Text2Quit, some messages come from a fictitious quit pal who offers social support (eg, Erika/Text2Quit). In other programs, users are paired with an actual quit buddy to interact via text and who supports their quit attempt [38].

##### Degree to Which the Program Will Be Tailored

A decision has to be made as to whether the program will run as a single generic program, with all users receiving the same content, whether there will be different versions (or protocols) for different types of users, or even whether individually tailored versions of the program may be offered. In general, creating extra protocols or tailoring to individual characteristics can be more expensive and therefore should be carefully thought through ahead of time. Tailored protocols may be considered because they can result in higher readership, higher message recall, perceptions of higher personal relevance, and in some cases, greater behavior change [39]. Some evidence exists to support the use of tailored text messaging programs [3,40]. Kharbanda et al [40] found it was helpful to add a child’s name to a text message about immunizations and follow-up vaccines, because it was more salient for parents. In another study, adolescent participants expressed that it would be more appealing if they were asked how they were feeling and the responses were tailored directly to them [34]. Logic can be built so that participants receive different message content based on their stage of change, readiness, and timing around key behaviors of interest (ie, quitting or birth of baby). This logic can be assigned from the outset, or can be modified based on responses to messages received by the program.

**Table 1:** Message examples based on approach.

Behavioral approach	Example message
1. Provide health information, advice, and tips, often tailored around user characteristics	Your baby is now 1 month old. Congrats! Remember to keep putting baby to sleep on his/her back to avoid SIDS.
2. Ask users to set goals	On what date will you quit smoking?
3. Provide opportunities for tracking progress	Track your exercise. Reply with the number of minutes you exercised yesterday.
4. Provide reinforcement for goals that are met	Congrats! You met your goal.
5. Offer reminders (eg, to take vitamins, to follow through with goals)	Your appointment is tomorrow.
6. Offer social support	Hi! My name is Mary. I’ve been through this and losing weight is tough. But if you stick with it, you’ll make it.

##### Supplemental In-Person Interaction

Models of mobile programs include programs that stand alone (eg, SmokefreeTXT) [41] or programs that supplement other existing programs such as face-to-face group counseling [42], phone counseling at quitlines, or those that supplement interactive websites. In cases in which texting supplements a larger program, mobile communication can be thought of as additional touch points to reinforce messaging from counseling sessions or Internet programs. To date, most text messaging programs for behavior change that have been evaluated have been stand-alone programs [16,17], and evidence to support their use as a supplemental platform (in addition to other in-person or mobile/Web-based components) is mixed [43,44].

##### Privacy Concerns

Text messaging is not a secure technology and, therefore, has risks associated with it for transmitting personal health information. This is especially a sensitive issue when programs will include personal health information and originate from a Health Insurance Portability and Accountability Act (HIPAA) covered entity, within the US context [7], or meet other health care-related privacy standards internationally. One way to make risks more transparent to participants is to disclose them in clear language at the time of signing up. Privacy risks are detailed in the terms and conditions of program use and explicitly state that text messaging is not secure, and that by signing up, the subscriber agrees to the risks. One example is how the NCI describes their terms of service online for the use of SmokefreeTXT [45]. A similar disclosure will be included in the roll-out of the Veteran Administration’s Annie text messaging system, which is currently being pilot tested and will perform a variety of functions including the collection of blood pressure readings [9].

#### Step 2d: Write the Message Library

The message library is a database of specific messages that will be sent to the user. Messages need to be written for each case supported by the program. Messages need to be 160 characters (including spaces) or less to be delivered as a single text message to a mobile phone, unless the users have smartphones. When necessary, messages may be split between 2 text messages to accommodate additional content. For an example of an existing message library, see QuitNowTxt message library [30]. Once written, a message library can be checked—by coding each message for its content—to see that it conforms to the planned communication objectives and behavioral techniques [26].

The following are some considerations when writing the message library.

##### Messages Can Take Many Forms

Messages can be based on many behavioral approaches including providing information or advice, asking users to track behaviors, providing feedback on goals, offering reminders, providing positive reinforcement, or providing social support (see Table 1 for examples). Chib et al [46] used texting to ask quiz questions about HIV transmission and testing. We recommend limiting each message to a single topic or actionable item.

##### Message Style

Assuming the source is a credible health entity, the language should fit the professional character of the organization and not be inclined to use slang, too many abbreviations (eg, “how r u doin?”), or informal punctuation (eg, “well done!!!!”). Our experience is that users find this type of language to be unprofessional coming from a credible health source. According to Ranney et al [34], even teens expressed that they did not like slang used in health messages.

##### Literacy Demands of Target Audience

Once the message library is drafted, check the literacy demands associated with the messages. This can be done using the Flesch-Kincaid Grade Level Test or Flesch Reading Ease test tool to determine the readability of the messages. In general, shorter words and sentences have lower literacy demands. Generally, for an adult audience, an 8th-grade reading level is considered the maximum recommended reading level [13], and in some cases, a lower reading level may be necessary. Further, with populations that may have low English literacy, but higher literacy in another language, a text messaging program can offer a translated version. For example, the program Text4Health provided parents with the option of switching the program language as follows: “To receive messages in Spanish, text ESPANOL” [40].

##### Mobile Phone or Social Media Integration

Given that mobile phone penetration is at 64% among adults in the United States [25], and there are up to an estimated 1.75 billion users globally [47], users are likely to be reading text messages on mobile phones. This means that text messages can be seamlessly linked to Web content to expand their content in the form of mobile Web pages, videos, audio, games, and social media. Increasingly, text messaging programs are being built in conjunction with mobile phone apps. The large-scale texting program, text4baby, now has an app that can be downloaded to support the text platform, as well as mobile Web pages offering additional content to supplement approximately 50% of the text messages [48]. However, because this adds additional development costs, it is important to study whether the addition of apps confers benefits to the end user. Additionally, social media provides another low-cost extension for an SMS intervention. In a weight-loss intervention for college students, the enhanced group received Facebook content reinforced with SMS content [44].

##### Build Automated Evaluation Into the Programming

The message library can be written to include periodic check-ins about the program’s success. These may take the form of surveying users about behavior change of interest (eg, “Text2Quit: Have you smoked a cigarette over the past 7 days? Reply 1 if yes or 2 if no”). These offer the opportunity to redirect program messaging for the user (eg, If reply is 1: “Sorry to hear you slipped; Here’s what you can do to get back on track.”), as well serve the purpose of providing valuable data on participant engagement and a program’s success [15]. Reminder text messages can be effective in prompting users to reply, in order to increase response rates for key program evaluation metrics. One consideration related to response rates is that there is likely to be more missing data with the increasing frequency of check-ins. Thus, the importance of higher response rates will need to be balanced against providing multiple opportunities to check-in with users and obtain updates on their status.

### Step 3. Pretesting the Text Messaging Program

Once the program is designed, we suggest building in a pretesting phase to solicit feedback on the program and the specifics of the message library. Based on feedback from this phase, revisions should be made to improve the program. Ideally, this process should be iterative and involve multiple rounds of revisions and feedback [49]. The following are ways to obtain feedback on the program.

Prior to Launch, Conduct Interviews With Target Audience Members to Test the Program and Sample Messages

In these interviews or focus groups, describe the program to potential users, show them the message library (or portions of it), and ask for feedback on specific messages (eg, see Ybarra et al [49]). Users might be asked to rate messages for tone, content, clarity, and persuasiveness. They could be asked to rewrite messages that are objectionable, unclear, or otherwise unsatisfactory. This might be done in-person, over the phone, by email, or through a message board on a website. To simulate receiving program text messages, part of the pretesting interview may include sending sample text messages to the users’ phone and asking for feedback on individual messages via SMS.

Launch Program for Pilot

There are a number of easy-to-use SMS platforms that provide the opportunity to set up a program using a Web-based interface that allows for the scheduling of text messages and provision of interactive surveys, keywords, and branching logic (targeted scheduling of messages and content). Some are free or low-cost programs like TextIt and Ez Texting, and are especially well-suited for use with a small group and for a limited period; however, many others exist and can be found highlighted in available resources like the mHealth Platform Compendium [50], the Text Messaging in Healthcare Research Toolkit [13], and the Mobile Messaging Toolkit [14]. Another low-cost method for piloting is to send out program messages manually to simulate the experience of an automated program. This can be done with a program like Google Voice, so that messages can be sent from a desktop.

Pilot Test the Program

After the program is up and running, feedback from users can be obtained by running a short pilot test, such as 2-4 weeks in length and with as few as 10-30 participants, and surveying users about their experience [15,49,51,52]. Surveys may be conducted by phone, on the Internet, or by SMS. Key areas to examine in the pilot test include the following: What is the user experience of being in the program? What about the program is most and least engaging? Is there anything confusing or annoying about the program? How is the message volume and timing? Did participants unsubscribe? Did participants change behavior? These issues can be addressed with both survey questions and qualitative (long-response) feedback.

In addition to survey data, computer records of program use, if available, reveal program engagement, and in some cases, behavior change [39,53-56]. Ideally, computer records will detail every instance of user interaction with the SMS system (complete with time and date stamp) and the nature of that engagement. It is important to understand how the program you choose to utilize collects user data and what data are available to you. For example, computer records can indicate at what point users text in for additional help and/or how they are doing in the program, assuming the program contains check-ins on behavior change (eg, Did you exercise today?).

Additionally, the keyword STOP, a standard keyword across programs for unsubscribing [13], provides a marker of program disengagement. To understand disengagement, we recommend using a 1-item survey at the point of unsubscribing. When users unsubscribe with the keyword STOP, it is sometimes possible to send a 1-item survey that asks for the reason(s) for unsubscribing. This could be completed as an open-text response or using a multiple-choice approach. Such feedback can be valuable and differentiate between users who stop because they have successfully changed their behavior and, therefore, no longer need the program (eg, “already quit”) and those who find the program unhelpful throughout (eg, “not helpful”)*.* If a user complains about receiving too many messages, this may be an opportunity to offer a low-dose version of the program to keep the user engaged and subscribed.

Test Alternate Versions of the Program

If possible, consider testing 2 (or more) versions of the program to evaluate users’ experiences on key decision variables (eg, program with and without a “buddy” protocol). Another option is to change the program over time while monitoring key metrics of success (eg, unsubscribe rates, engagement rates, levels of behavior change). Once the program is launched, it is important to be aware of different dissemination processes (including public Web-based dissemination like that done for the NCI’s texting programs) so as to not contaminate any randomized controlled trials in which use of the program is assigned.

Iterate

Like other health behavior interventions, these text messaging programs should be revised. Based on feedback from users collected through surveys and computer records of use, the programs should be constantly improved, and new versions deployed for use.

## Discussion

Based on the authors’ experiences of designing, developing, trialing, and implementing text message behavior change programs, it is important to have a good design process. Perhaps one of the most important factors is to be flexible and responsive to the input and feedback of your target audience: if they do not enjoy the program they may disengage.

## Abbreviations

CDC: Centers for Disease Control and Prevention
HIPAA: Health Insurance Portability and Accountability Act
mhealth: mobile health
NCI: National Cancer Institute
NHS: National Health Service
SMS: short message service
VHA: Veteran’s Health Administration
WHO: World Health Organization
